# A new Oligocene-Miocene tree from Panama and historical *Anacardium* migration patterns

**DOI:** 10.1371/journal.pone.0250721

**Published:** 2021-06-02

**Authors:** Oris Rodríguez-Reyes, Emilio Estrada-Ruiz, Camila Monje Dussán, Lilian de Andrade Brito, Teresa Terrazas

**Affiliations:** 1 Instituto de Ciencias Ambientales y Biodiversidad, Universidad de Panamá, Estafeta universitaria, Panamá, Panamá; 2 Smithsonian Tropical Research Institute, Panama City, Panamá; 3 Departamento de Zoología, Laboratorio de Ecología, Escuela Nacional de Ciencias Biológicas, Instituto Politécnico Nacional, Ciudad de México, México; 4 Departamento de Botânica, Instituto de Biociências, Universidade de São Paulo, Rua do Matão, Cidade Universitária, São Paulo, São Paulo, Brazil; 5 Departamento de Botânica, Centro de Ciências da Saúde, Universidade Federal do Rio de Janeiro, Ilha do Fundão - Rio de Janeiro, Rio de Janeiro, Brazil; 6 Instituto de Biología, Universidad Nacional Autónoma de México, Ciudad de México, Mexico; University of California, UNITED STATES

## Abstract

Migration of Boreotropical megathermal taxa during the Oligocene and Miocene played a key role in assembling diversity in tropical regions. Despite scattered fossil reports, the cashew genus *Anacardium* offers an excellent example of such migration. The fossil woods described here come from localities in Veraguas, Panama mapped as Oligocene-Miocene. We studied, described, and identified two well-preserved specimens using wood anatomical characteristics and completed extensive comparisons between fossil and extant material. The studied fossil woods share several diagnostic features with the modern *Anacardium* genus, including large solitary vessels, large intervessel-pitting, a simple vessel-ray pitting pattern, and mostly 1–3 seriate rays with large rhomboidal solitary crystals. We propose a new fossil species named *Anacardium gassonii* sp. nov., that adds an essential piece to the understanding of the historical biogeography of the genus. In addition, our findings confirm previous interpretations of this species’ migration from Europe to North America and its crossing through Panama, leading to subsequent diversification in South America. This discovery provides an important link to the historical migration patterns of the genus, supporting the notion of an Eocene migration to the Neotropics via Boreotropical bridges, as well as an Oligocene-Miocene crossing of Central America followed by diversification in South America.

## Introduction

The Anacardiaceae family has approximately 80 genera and 900 species, represented by trees, shrubs, and some woody climbers; the family is widely distributed in tropical and subtropical areas as well as in warm-temperate regions [1]. The extensive fossil records of Anacardiaceae worldwide make this family an excellent example for biogeographical studies. There are approximately 80 reported fossil woods associated with the Anacardiaceae family [e.g., 2–8]. Most of these records are from South America and Asia [9].

To date, there have been a few reports of fossil Anacardiaceae in Panama based on permineralized fruits and silicified woods. The oldest reported fossil remain is an endocarp identified as *Dracontomelon* L., which was recovered from the Eocene Tonosí Formation [10]. Three Miocene Spondioideae fruits from the Cucaracha Formation in the Panama Canal, identified as *Spondias rothwellii*, *Dracontomelon montesii*, and *Antrocaryon panamensis* [11], document the significance of the Spondioideae in early Miocene Panama forests.

Reports of Anacardiaceae based on silicified woods only include a large trunk from the Miocene Santiago Formation. The *Llanodelacruzoxylon sandovalii* Rodríguez-Reyes, Estrada-Ruiz et Gasson was found in Llano de la Cruz, Veraguas [12]. *Burseroxylon* fossil woods collected in the vicinity of Ocú have also been noted, but these samples could not be assigned to either the Anacardiaceae or Burseraceae family [13].

While the Anacardiaceae family is well represented in the fossil record, the cashew genus *Anacardium*, currently restricted to Central and South America, accounts for only a few reliable fossil discoveries. The oldest fossilized remains of this genus were found on permineralized endocarps from the Middle Eocene Messel Formation. The specimen represents the first fossilized cashew with a preserved hypocarp, which unequivocally demonstrates that *Anacardium* was once native in Europe [14].

**Fig 1:**
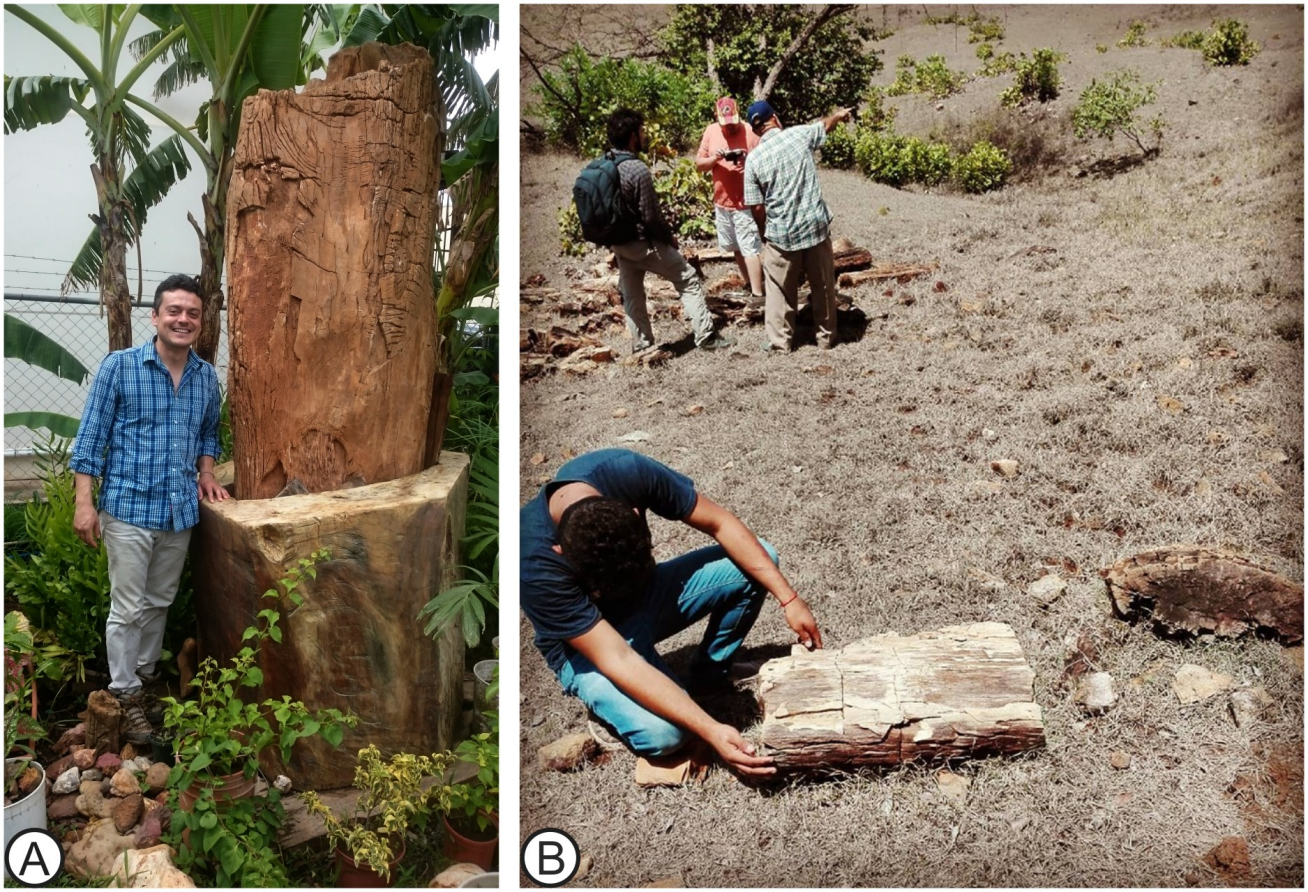
MUPAN-STRI 44071 and context of the field area. (A) Image of *Anacardium gassonii* taken in the field (B) Gullies in Los Boquerones farm in Veraguas, where several specimens were collected, including MUPAN-STRI 44051.

Here we add a key piece to the *Anacardium* biogeography map by documenting one of the largest fossil trunks reported from Panama, and probably Central America, to date (Fig 1). The fossil woods share most diagnostic features with modern *Anacardium* species. This new record sheds light on the biogeographical history of cashew nuts and their establishment in Central and South America since the Paleogene.

## Materials and methods

### Sampling

**Fig 2:**
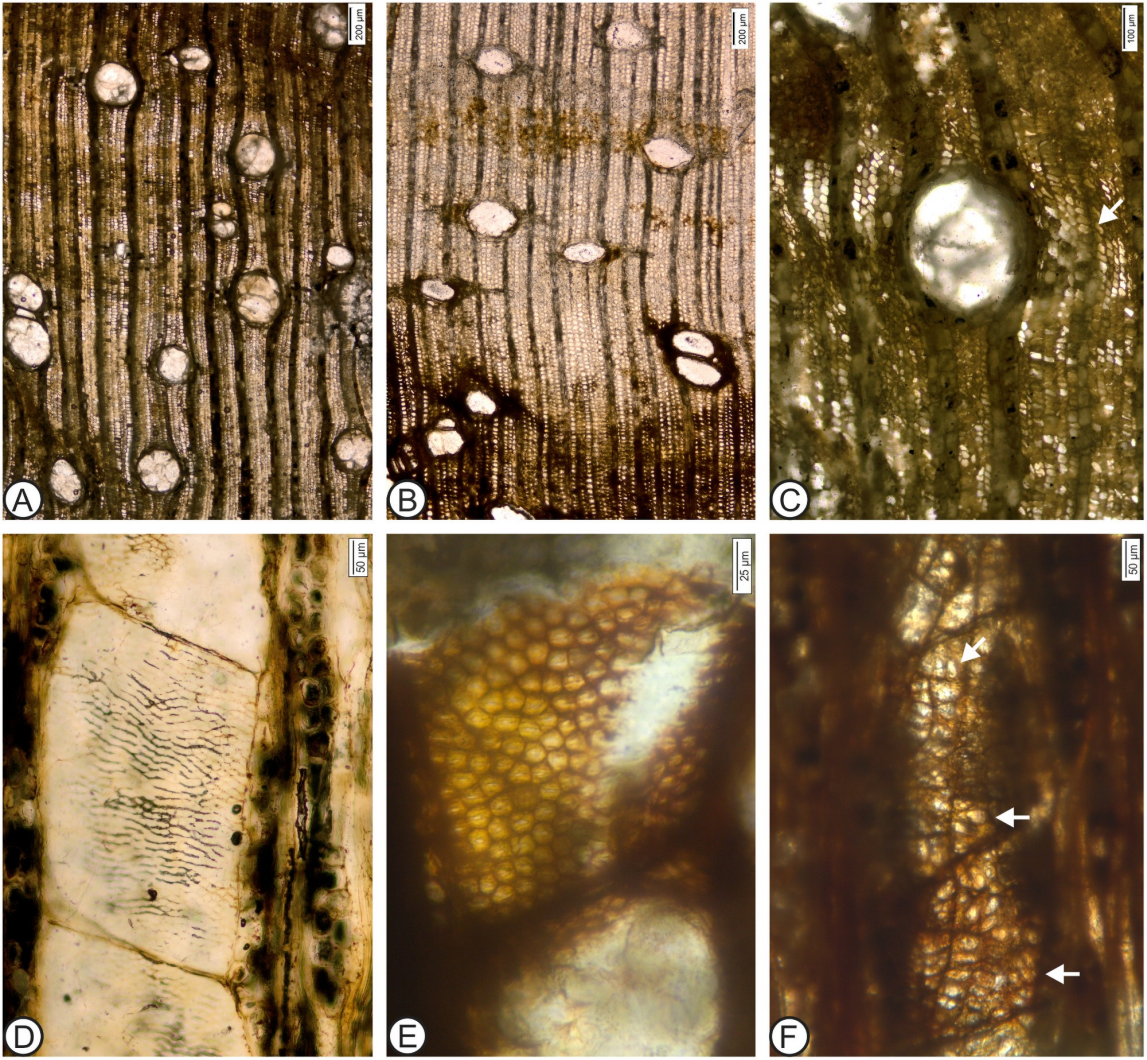
*Anacardium gassonii* Rodríguez-Reyes, Estrada-Ruiz et Terrazas sp. nov. A, C-F, MUPAN-STRI 44071; B, MUPAN-STRI 44051. (A) Growth ring boundaries absent; wood diffuse-porous (TS). (B) Solitary vessels and aliform parenchyma (TS). (C) Detail of the aliform parenchyma (arrow) (TS). (D) Simple perforation plate (TLS). (E) Intervessel pits alternate, polygonal and crowded (TLS). (F) Vessel–ray parenchyma pits with much reduced borders, round to horizontally elongated (TLS).

Samples of MUPAN-STRI 44071 and MUPAN-STRI 44051 (Fig 1) were donated by Mr. Carlos Sandoval, a local farmer who has provided several samples for analysis. Both specimens were accessioned in the Smithsonian Tropical Research Institute repository, Panama (https://biogeodb.stri.si.edu/jaramillosdb/web/fossils/). We highlight that MUPAN-STRI 44071 was a remarkably large specimen, with a perimeter of ~2.5 m and a preserved length of nearly 7 m. The trunk was found in Los Boquerones, Veraguas, where we also collected the hand sized STRI 44051 specimen (latitude 08° 13’ 46.4" N: longitude 80° 51’ 45.1" W). Unfortunately, we do not have measurements for the total preserved length of the original trunk (Figs 1A and 2B) because a few pieces were extracted and sold (personal communication, Mr. Carlos Sandoval, 2019).

### Geological setting

The wood specimens described here come from gullies in Boquerones, Veraguas, Panama. The radiometric ages of the geological units exposed in this area are unknown; however, the units have been mapped as Oligocene-Miocene [15–17]. In a recent visit to Los Boquerones, we did not observe much exposure to the related geologic unit. We have explored the surrounding areas identified as part of the Miocene Santiago Formation, but we restrain from inferring that the woods analyzed here are from the same formation. Further detailed geologic mapping is needed in this area to confirm these conclusions.

### Fossil specimen preparation and identification

Petrographic thin sections of fossil material were prepared in transverse (TS), radial longitudinal (RLS), and tangential longitudinal (TLS) sections. Sections were ground to a thickness of ~30 μm, mounted on glass slides using EpoFix resin, and coverslips were affixed with a UV-curable acrylates gel. The material was observed and imaged using an Olympus BX53 and an SC100 digital camera with a 10.5 Mpix CMOS sensor and a Zeiss AXIO Zoom V16; the material was then photographed with an AxioCam MRc5 camera.

The fossil woods were compared with available images of modern and fossil woods from the Inside Wood Database (IWD; insidewood.lib.ncsu.edu) [18] and literature [e.g. 8, 12, 19–22]. We also made a plate of modern *Anacardium* micromorphology slides from the collection at Instituto de Biología, UNAM, México.

### Geographic distribution

The geographic distribution was plotted using QGIS software [23] and using free vector map data from Natural Earth [24]. For taxon occurrences, we included information from the location of *A*. *gassonii* (described here) and *A*. *germanicum* [14]. Modern distribution data were obtained from [25].

### Nomenclature

The electronic version of this article in Portable Document Format (PDF) in a work with an ISSN or ISBN will represent a published work according to the International Code of Nomenclature for algae, fungi, and plants, and hence the new names contained in the electronic publication of a PLOS ONE article are effectively published under that Code from the electronic edition alone, so there is no longer any need to provide printed copies. The online version of this work is archived and available from the following digital repositories: PubMed Central, LOCKSS.

### Systematic palaeobotany

**Order** Sapindales Jussieu ex Berchtold & J. Presl

**Family** Anacardiaceae Lindley

**Genus** *Anacardium* Linnaeus

**Fig 3:**
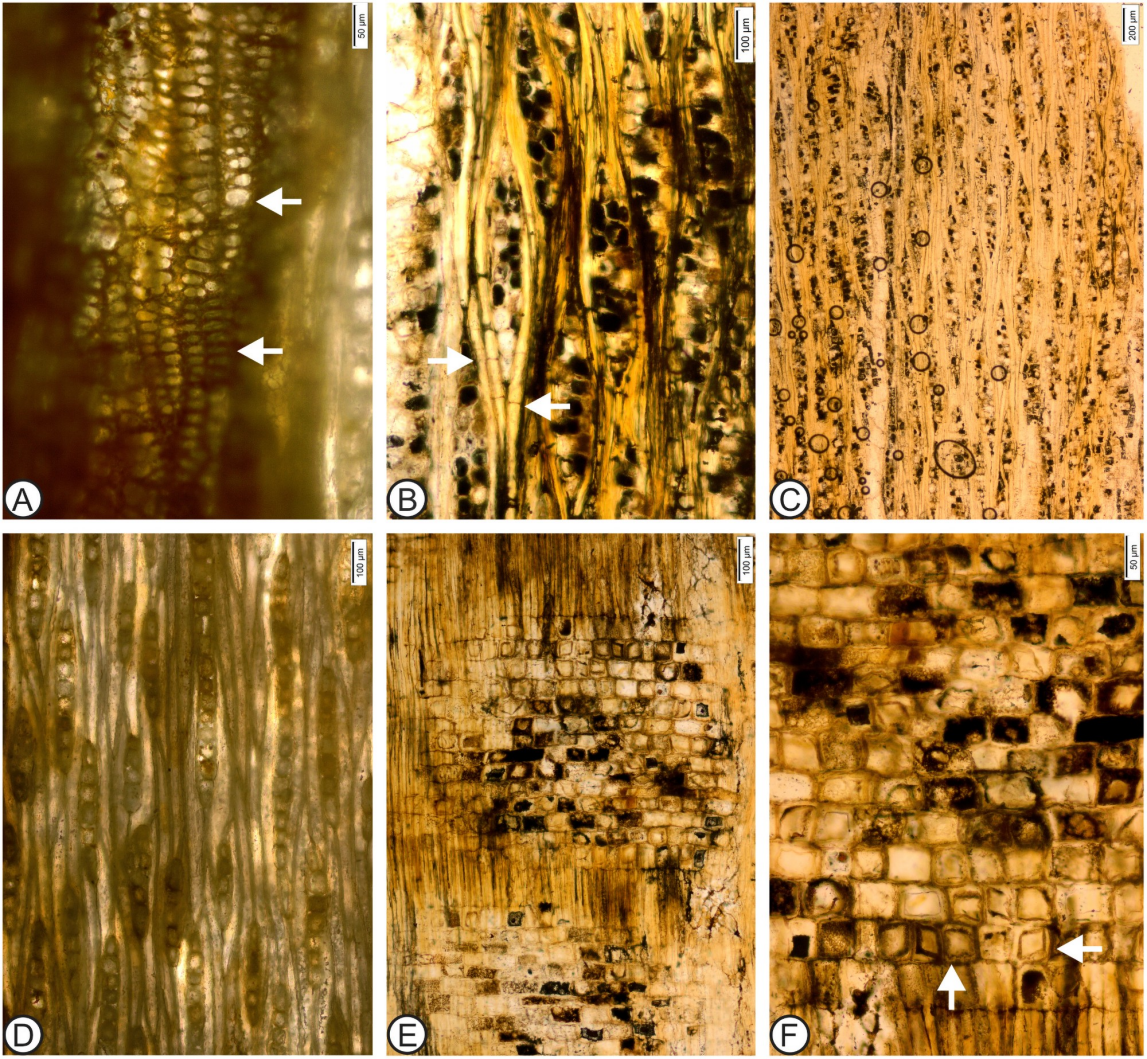
*Anacardium gassonii* Rodríguez-Reyes, Estrada-Ruiz et Terrazas sp. nov. B, C, E, F, MUPAN-STRI 44071; A, D, MUPAN-STRI 44051. (A) Vessel–ray parenchyma pits with much reduced borders, elongated and slightly in palisade (RLS). (B) Septate (arrows) and non-septate fibers (TLS). (C) Rays with 1–3 cells wide (TLS). (D) Rays mostly uniseriate (TLS). (E) Heterocellular ray (RLS). (F) Prismatic rhomboidal solitary crystals present in the procumbent and square ray cells (RLS).

Species ***Anacardium gassonii*** sp. nov. Rodríguez-Reyes, Estrada-Ruiz et Terrazas (Figs 2 and 3).

**Specific Diagnosis**: Growth rings absent, wood diffuse porous. Vessels solitary combined with a few short radial multiples of 2 (–3). Perforation plates exclusively simple. Intervessel pits alternate, polygonal and large; vessel–ray parenchyma pits with much reduced borders, round and horizontally elongated to slightly in palisade. Mean tangential vessel diameter > 200 μm. Non-septate fibers combined with occasional septate fibers. Axial parenchyma lozenge-aliform and vasicentric. Rays 1–3 (–4) cells wide.

**Holotype**: MUPAN-STRI 44071

**Paratype**: MUPAN-STRI 44051

**Locality**: Los Boquerones, Veraguas, Panamá

**Stratigraphic position and age**: Oligocene-Miocene

**Etymology**: Named in honor of Dr. Peter Gasson for his valuable contributions to the study of wood anatomy.

### Detailed description

Description based on two samples of wood. Growth rings absent; wood diffuse porous. Vessels solitary combined with a few short radial multiples (32%) of 2 (–3) (Fig 2A–2C); vessel outline oval to rounded (Fig 2A and 2C). Exclusive simple perforation plates (Fig 2D and 2E). Intervessel pitting alternate, polygonal, and large (mean pit diameters range 11 to 15 μm) (Fig 2E); vessel–ray parenchyma pits with much reduced borders, round to horizontally elongated to slightly in palisade (Figs 2F and 3A). Mean tangential vessel diameter 261 μm (range 160–310 μm); mean vessel frequency 3 mm^-2^ (range 2–6 mm^-2^) (Fig 2A–2C). Mean vessel element length 416 μm (range 284–545 μm) (Fig 2D). Non-septate fibers and occasional septate fibers, with thin to medium walls, mean thickness 2.7 μm (range 1.2–4.17 μm) and lumen with 16.7 μm (range 12–22.7 μm) (Fig 3B–3D). Axial parenchyma lozenge-aliform and vasicentric (Fig 2A–2C). Parenchyma strands mostly 3–8-celled. Rays 1–3 (–4) cells wide (Fig 3B and 3D). In the paratype, rays are 1–2 seriate (Fig 3D). Mean ray height is 555 μm (range 423–821 μm) and 13 cells (range 7–20 cells); spacing with means 7–12 per mm (Fig 3C), composed of mixed cells throughout the ray body. Occasionally the rays are composed exclusively of square and upright cells (Fig 3E and 3F). Abundant prismatic rhomboidal solitary crystals present in the procumbent and square ray cells (Fig 3F). Tyloses present.

### Comparative remarks

We conducted several searches using the Inside Wood Database. The most restrictive search was as follows: wood diffuse-porous (5p), vessels in tangential bands absent (6a), vessels in diagonal and/or radial pattern absent (7a), vessels in dendritic pattern absent (8a), exclusively solitary vessels absent (9a), vessels in radial multiples of 4 or more common absent (10a), vessel clusters common absent (11a), simple perforation plates (13p), intervessel pits alternate (22p), intervessel pits large (27p), vessel-ray parenchyma pits with much reduced borders to simple: pits rounded or angular (31p), mean vessel tangential diameter > 200 μm (43p), axial parenchyma aliform (80p), exclusively uniseriate rays absent (96a), larger rays commonly > 10-seriate absent (99a), all rays procumbent absent (104a), prismatic crystals present (136p), prismatic crystals in upright and/or square ray cells (137p), tree (189p) with 0 allowable mismatches. We obtained 23 results, all belonging to three families: Anacardiaceae, Moraceae and Urticaceae. We ruled out the Moraceae genera because of the common occurrence of laticifers. Although *Streblus glaber* do not show laticifers, this species can also be distinguished from the fossil sample because it possesses abundant sclerotic tyloses, banded parenchyma, and sheath cells. Our fossil wood specimen is distinct from the Urticaceae results based on the occurrence of marginal bands of parenchyma, sheath cells, and the stronger winged parenchyma pattern. The listed results included several Spondioideae genera; therefore, we completed a comparison with available information in the IWD and the literature, e.g., [18–20] and the IWD, which is compiled in Table 1.

**Table 1:** Comparative table between *Anacardium gassonii* and selective traits of Spondioideae and two Anacardioideae genera.

Genera	GR	DP/R	VA	PP	IVP (μm)	V-R P	MVD (μm)	VF (mm^2^)	SF	MVEL (μm)	AP	PS	RW	RS (mm)	RC	RComp	PC	SB	HT
*Allospondias*	N	DP	N	S	alt;14	RB	165	2	N	431	V	?	3	4	Y	1, 2–4 up/sq	C up/sq, pro	N	M
*Antrocaryon*	Y	DP	N	S	alt; o-p; 5–11	RB	107–206	4–6	Y	499–624	SC, NV	4–9	3 (2–5)	4–6	Y	1–5 up/sq	N	N	L
*Buchanania*	Y/N	DP	N	S	alt; p; 10	RB	59–351	1–15	N (Y)	190–938	SC, V, LA, C	2–8	2 (2–4)	5–9	Y	ho	C up/sq	Y	L-M
*Campnosperma*	N	DP	Dv	S(Sc)	alt; p; 10	RB; H	44–181	16–46	N (Y)	359–1572	R	2–5	2 (1–3)	3–5	Y (N)	ho; 1–2 up/sq	N	Y	L
*Choerospondias*	Y	R	Dv	S	alt; p; 13	RB; H	86–319	?	Y	179–653	SC, NV	3–9	2–5	3–5	Y	1–2 up/sq	C up/sq	N	M
*Cyrtocarpa*	N(Y)	DP	N	S	alt; p; 9	RB; H	23–122	36–72	Y	272–613	SC	4–9	4 (3)	3–5 (4)	Y	1–4 up/sq	C up/sq	N	S
*Dracontomelon*	N	DP	N	S	alt;p; 12	RB; A	100–219	2–4	Y	260–626	V, LA, U	5–17	4 (2–4)	4–6	N	ho	C pro	N	L
*Haematostaphis*	N	DP	N	S	alt;p; 10	RB; A	82–202	5–11	Y	234–604	V, LA	4–8	4 (2–4)	6–9	N	ho; mix	C pro	N	S
*Harpephyllum*	Y	DP	N	S	alt; o; 11	RB; A	55–121	12–22	Y	204–472	SC	2–6	2–9	6–8	Y	1 up/sq	C pro	N	L
*Koordersiodendron*	N	DP	N	S	alt; p;12	RB; H	83–204	4–8	Y	266–867	SC, V	3–11	2–3 (1)	4–8	Y	1 up/sq	C up/sq	N	L
*Lannea*	N(Y)	DP	N	S	alt; p; 11	RB; P	38–210	5–18	Y	300–876	SC, NV	2–4	2–5	3–6	Y	ho; mix	C up/sq	Y	M (S)
*Operculicarya*	N	DP	N	S	alt; p; 7–10	RB; P -H	50–100	5–20	Y	?	SC	3–4; 5–8	1–3; 4–10	4–12	Y	1–4 up/sq	N	N	S
*Pleiogynium*	N (Y)	DP	D	S	alt; o; 9	RB; P	89–215	8–11	Y	296–745	NV	3 to 7	3–7	4–8	Y	1–2 up/sq	C up/sq	N	L
*Poupartia*	N	DP	N	S	alt; p; 11	RB; P	56–165	5–25	Y	186–719	SC	6 to 8	2–5	3–8	Y	ho; 1–2 up/sq	C pro	N	S
*Pseudospondias*	N	DP	N	S	p; >10	RB; P	100–200	5–20	Y, N	350–800	V, LA	3–4 (5–8)	1–3	4–12	N	1–4 up/sq	C up/sq	N	L
*Sclerocarya*	N (Y)	DP	N	S	alt; p; 10	RB; P	45–220	9–17	Y	88–833	SC	3–7	4 (2–5)	5–7	Y	1–4 up/sq	C up/sq	N	S-M
*Solenocarpus*	N	DP	N	S	alt; p; 10	RB; H-V	100–200 (>200)	<5	Y	350–800	V	3–4; 5–8	4–10	4–12	Y	2–4 up/sq	C up/sq	N	L
*Spondias*	N	DP	N	S	alt; p; 14	RB; A-H	53–420	3–11	Y	167–957	LA, V	3–8	2–7	2–4	Y	1–4 up/sq	C up/sq	N	L-M
*Tapirira*	N	DP	N	S	alt; o-p; 12	RB; P	76–237	7–16	Y	560–890	SC	4–8	3–8	5–7	Y	2–4 up/sq	C pro	N	L
*Anacardium*	N	DP	N	S	alt; p; 11–15	RB;R-A;	22–330	<10	N (Y)	270–570	LA, V, C	2–8	1-2(3)	4–13	N	2–4 up/sq; ho	C pro	Y(N)	L-S
***Anacardium gassonii***	**N**	**DP**	**N**	**S**	**alt; p; 11**	**RB; R-H; P**	**270**	**3**	**N (Y)**	**416**	**LA, V**	**3–8**	**1–3 (4)**	**7–12**	**N**	**mix; ho**	**C up/sq (pro)**	**N**	**L**

GR = growth rings; DP/R = wood diffuse porous/wood ring porous; VA = vessel arrangement. PP = Perforation plates; IVP = intervessel pitting pattern and mean size; V-R P = vessel-ray pits pattern; MVD = mean vessel diameter; VF = vessel frequency. SF = septate fibers; MVEL = mean vessel element length; AP = apotracheal parenchyma; PS = parenchyma strands length; RW = ray width in number of cells. RS = ray spacing; RC = Ray cells; RComp = Ray composition; PC = Prismatic crystals; SB = silica bodies. HT = habit tree. Character states: N = no Y = yes; D = diagonal arrangement. v = variable state; S = simple perforation plates; Sc: scalariform perforation plates; alt = alternate intervessel pits; o = oval; P = polygonal shape; RB = reduced borders. H = horizontally elongated; A = angular vessel-ray pits; P = pits in palisade; V = vasicentric parenchyma; SC = scanty parenchyma; NV = narrow vasicentric; LA = lozenge aliform; U = unilateral parenchyma; WA = winged aliform parenchyma; up/sq = upright/square ray cells; ho = homocellular cells; mix = mixed cells throughout the ray; C up/sq = crystals in upright/square cells; C pro = crystals in procumbent ray cells; M = medium trees; L = large trees; S = small trees.

We also revised comprehensive surveys of Anacardiaceae mostly of the old Continent, e.g., [26, 27]. We included a few of these genera in Table 1. Genera such as *Bouea* and *Gluta* could be distinguished because of the occurrence of marginal bands of parenchyma, features absent in the fossil. *Cotinus* has ring-porous wood and helical thickenings. Woods of *Drimycarpus* possess aliform strong parenchyma. *Holigarna* has small vessels, abundant winged-aliform parenchyma and druses present. Radial canals are present in *Melanorrhoea*, *Parishia*,*Swintonia*, *Euroschinus*, *Melanochyla*, *Pentaspadon* and *Toxicodendron*, feature not observed in *A*. *gassonii*. *Nothopegia* is distinct because of very abundant aliform, confluent and banded parenchyma. *Parishia*, *Rhus* and *Pistacia* can be distinguished from the fossil because of wood ring porous, growth rings present and helical thickenings.

*Semecarpus* woods have a strong winged-aliform pattern and wider rays compared to *A*. *gassonii*. Also [28], reported that *Rhus*, *Cotinus* and *Pistacia* have oblique to dendritic latewood vessel distribution and narrow fibers. Finally, *Mangifera* sp. shows parenchyma bands not observed in the fossils.

## Discussion

### Comparison with *Anacardium* species

We revised the IWD and literature [19, 20] to further study the *Anacardium* genus and develop a more rigorous analysis of this fossil. A summary of quantitative features is presented in Table 2, where we note differences and similarities when compared to the species available in the literature.

**Table 2:** Comparative table of *Anacardium* modern species available in the literature compared to *Anacardium gassonii*.

*Species*	RM	SPP	MTVD (μm)	VF (mm ^2^)	IVP (μm)	V-R P	VEL (μm)	T	SF	PP	PS	RW (number of cells)	RS	PC
*Anacardium corymbosum*	2–3 (6–17)	excl	50–100	5–14	7–10	MRB	<350 (-800)	Scl	N	V, LA, C	2–8	3	4–12 (>12)	N
*A*. *excelsum*	2–3 (5–8)	excl	100-200(>200)	1–5	>10	MRB, H- V	350–800 (<300)	Y	N (Y)	V, LA, C	2, 3–4	1–3	4–12	Y
*A*. *giganteum*	2–4,	excl	100–200	1–5	>10	MRB, H, V	350–800	Y	N (Y)	V (WA)	2, 3–4, 5–8	1	4–8	N
*A*. *humile*	2 sizes	excl	<50 (50–100)	5–14	7–10 (>10)	MRB	<350	Y	N	V (WA)	3–4, 5–8 (2)	1–3	9–13	N
*A*. *nanum*	10–18 (2 sizes)	excl	50–100	5–20	7–10 (>10)	MRB	<350	N	N	V (WA)	3–4, 5–8 (2)	1–3	4–12	N
*A*. *occidentale*	2–4 (6–17)	excl	100-200(>200)	5–14	>10	MRB	350–800	N	N	V, LA, C	2, 3–4	1–3	9–13	N
*A*. *parviflorum*	2–4	excl	100–200)	4–7	7–10	MRB	?350	N	Y	Sc, V	2–8	1	4–8	N
*A*. *spruceanum*	2–4	excl	100–200	1–5	7–10 (>10)	MRB, H- V	350–800	Y	N	V, LA (C)	2, 3–4	1	4–8	N
***A*. *gassonii***	**2–3**	**excl**	**160–310**	**2–6**	**11–15**	**MRB, H-V**	**284–545**	**N**	**N(Y)**	**V, WA, LA**	**3–8**	**1-3(-4)**	**7–12**	**Y**

RM = radial multiples; SPP = simple perforation plates; MTVD = mean tangential vessel diameter; VF = vessel frequency; IVP = intervessel pits size; V-R P = vessel ray pits; VEL = vessel element length; T = tyloses abundant; SF = septate fibers; PP = parenchyma pattern. PS = parenchyma strand length; RW = ray width in number of cells; RS = ray spacing. PC = prismatic crystals. Character state codes: excl = exclusively; MRB = vessel-ray pits with much reduced borders; H = horizontally elongated pits; V = vertical pits. Scl = sclerotic tyloses; Y = yes; N = no; V = vasicentric parenchyma; LA = lozenge aliform parenchyma; C = confluent parenchyma; WA = winged aliform; Sc = scalariform parenchyma.

A few qualitative differences were noted when we compared the fossils with several species of the genus. For example, two distinctive vessel size categories can be observed in *A*. *corymbosum*, *A*. *humile*, *A*. *nanum*, and *A*. *occidentale*. In addition, sclerotic tyloses occur in *A*. *corymbosum*, while woods of *A*. *giganteum* and *A*. *parviflorum* have only uniseriate rays. The feature that most strongly distinguishes this new fossil species from most of the *Anacardium* is the abundance of silica bodies in the ray cells. The sole species that lacks silica bodies and has abundant large prismatic crystals, as in the case of the study fossil *A*. *gassonii*, is *A*. *excelsum*.

**Fig 4:**
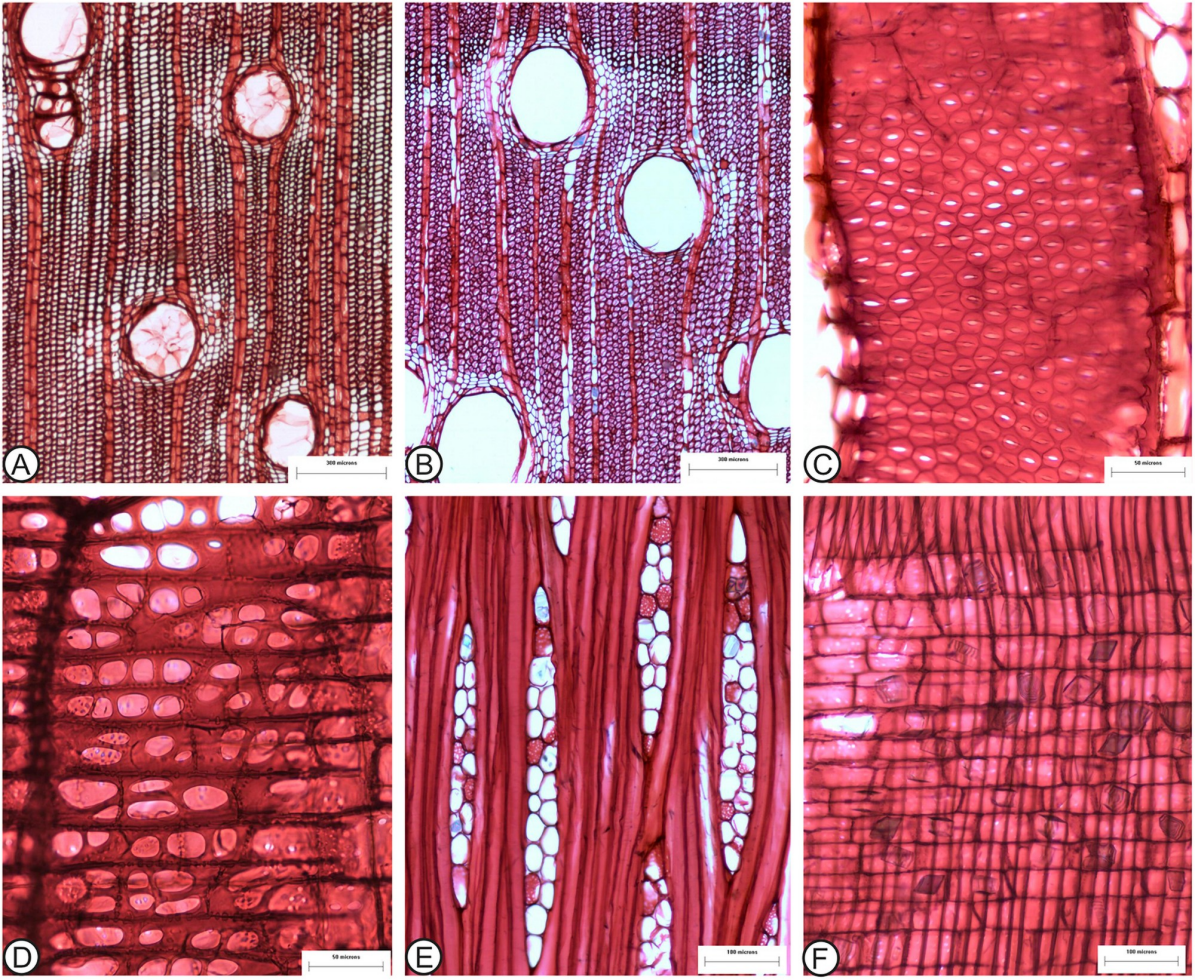
*Anacardium excelsum* wood anatomy. A, C, D, F MAD4433; B, E, MAD11551 (A) Growth ring boundaries absent, wood diffuse-porous (TS). (B) Large vessels; lozenge-aliform parenchyma (TS). (C) Intervessel pitting alternate, large, and polygonal (TLS). (D) Vessel-ray pits with much reduced borders, horizontally elongated and in palisade (RLS). (E) Rays 1–2 seriate, non septate fibers (TLS). (F) Rays composed of upright and square cells with abundant large rhomboidal crystals (RLS).

The new fossil species described here shows a strong resemblance to *Anacardium excelsum* (Fig 4), sharing the following diagnostic features: the absence of growth rings; solitary vessels with a few radial multiples (Fig 4A and 4B); vessel outline oval to round (Fig 4B); simple perforation plates; alternate, polygonal, and large intervessel pitting (Fig 4C); vessel–ray parenchyma pits with highly reduced borders, round to horizontally elongated (Fig 4D); mean tangential vessel diameter > 200 μm (Fig 4A and 4B); axial parenchyma lozenge-aliform, and vasicentric (Figs 3B and 4A); rays mostly 1–3 cells wide (Fig 4E); mean ray spacing 7–12 per mm (Fig 4E); and composed and abundant large prismatic rhomboidal solitary crystals present in the procumbent and square ray cells (Fig 4F). In a few specimens of *A*. *excelsum* reported in the literature, the vessels are smaller, the inclination of the perforation plates is higher, and parenchyma strands can be shorter. After a detailed comparison, we confidently assign this fossil wood to the *Anacardium* genus, and we highlight its similarities to the modern *Anacardium excelsum* species. Trees of this species can attain 2 m in trunk diameter and reach 40 m in height; today they are commonly present in Panama, mainly in the Pacific slope. These trees are especially abundant along streams, and a few large individuals can inhabit mature forests. The species adapts well to disturbed areas (Pérez and Condit, n.d.).

The discovery of these fossils supports the notion that the genus *Anacardium* was present in Central America since the Oligocene-Miocene, suggesting that *A*. *gassonii* could represent the ancestral species for modern neotropical *Anacardium* species. This assertion may be supported by future phylogenetic analysis for the genus.

### Comparison with fossil taxa

We conducted several searches in the Inside Wood Database (http://insidewood.lib.ncsu.edu; [17]), using the fossil wood menu. Most of the results are related to Anacardiaceae, but a few ones are assigned to other families that include Euphorbiaceae, Clusiaceae, Combretaceae and Leguminosae. We can rule out all *Euphorbioxylon* (emmended by [29]), because these possess rays up to five cells wide, rays 1 mm high and all fibers are non-septate fibers. *Guttiferoxylon* and *Symphonioxylon* have banded parenchyma and all non-septate fibers. *Glutoxylon* has radial canals all fibers are non-septate.

Two large genera we compared with *A*. *gassonii* were *Terminalioxylon* and *Leguminoxylon*. We can distinguish this fossil from *Terminalioxylon* because several species have axial canals (e.g., *T*. *annamense*, *T*. *coriaceoum*, *T*. *edwardsii*, *T*. *fezzanense*, *T*. *traumaticum*), vessel-ray pitting similar to minute intervessel pits (e.g., *T*. *chowdhurii*, *T*. *intermedium*, *T*. *kratiense*, *T*. *matrohense*, *T*. *panotlensis*, *T*. *primigenium*, *T*. *speciosum*), exclusively uniseriate rays (e.g., *T*. *annamense*, *T*. *chowdhurii*, *T*. *edwardsii*, *T*. *erichsenii*, *T*. *panotlensis*, *T*. *felixii*, *T*. *sahnii*, *T*. *siwalicus*, *Terminalioxylon sp*, *T*. *sulaimanense*, *T*. *welkitii)*, banded parenchyma (e.g., *T*. *annamense*, *T*. *cf naranjo*, *T*. *chowdhurii*, *T*. *coromandelinum*, *T*. *doubingeri*, *T*. *felixii*, *T*. *fezzanense*, *T*. *geinitzii*, *T*. *pachitanensis*, *Terminalioxylon sp*, *T*. *sulaimanense*, *T*. *tertiarum*).

We ruled out *Leguminoxylon* because of occurrence of banded parenchyma (e.g., *L*. *dindense*, *L*. *grossei*, *L*. *aff*. *schoelleri; L*. *acacia*, *boureaui*, *L*. *medarbaense*, *L*. *schenkii*, *L*. *submenchikoffii*, *L*. *aff*. *cystisus*); the combination of semi-ring porous wood and marginal parenchyma (e.g., *L*. *ersanense*, *L*. *ouniangaense*, *L*. *sahabiense*) [30]; rays storied (*L*. *aethiopicum*, *L*. *welkitii*); homocellular rays (*L*. *aff*. *genabens*, *L*. *bonneti*, *L*. *lefranci*, *L*. *menchikoffi*, *L*. *monodi*, *L*.*schenkiii*,*L*. *submenchikoffii*, *L*. *tamendjeltense*, *L*. *teixeirae*, *L*. *zemletense*) and vessels with a diagonal arrangement (*L*. *ligerinum*, *L*. *paraersanense)*.

We surveyed selected Anacardiaceae fossil genera from the results of the Inside wood Database and key literature. These included *Mangiferoxylon*, that possess abundant banded parenchyma and paratracheal winged-aliform parenchyma, traits absent in *A*. *gassonii; Swintonioxylon* have banded parenchyma and radial canals [31]; *Holigarnoxylon* has smaller vessels (96–197 μm) and considerably shorter rays (277–512 μm) compared to the new fossil species. Alsosolitary primastic crystals are not observed. Additionally, in the images from Shukla and Mehrotra [31], we can observe vessel-ray pits that are round in shape, whereas the ones in *A*. *gasssonii* are round and horizontally elongated to slightly in palisade; *Pistacioxylon* is characterized by vessel clusters common, radial canals and helical thickenings, characters not observed in the new fossil wood discussed here.

We compared *A*. *gassonnii* to another new Anacardiaceae fossil genus from the Santiago Formation in Panama, *Llanodelacruzoxylon sandovalii* Rodríguez-Reyes, Estrada-Ruiz & Gasson. This wood has axial parenchyma apotracheal diffuse and scanty paratracheal and the vessel-ray pits are smaller and round, whereas the new fossil wood discussed here has vessel-ray pits tend to be horizontally elongated and slightly in palisade.

### Fossil record of *Anacardium* and comparison with other *Anacardium* fossils

The fossil record of *Anacardium* is incomplete. To date, the oldest fossil of the genus was recovered from the Eocene Messel flora in Germany and consists of fruits with attached hypocarps [14]. In their work, Manchester et al. [14] provide a comprehensive survey of other *Anacardium* fossil reports, which include several permineralized fruits from Colombia, Ecuador, and Peru. They concluded that the most reliable report is a fossil fruit found in Messel. Other fossils related to *Anacardium* include pollen grains from the Middle Miocene Salto de Tequendama, Colombia [32] and others from Malaysia, which were reported as “Tertiary” age [33].

Regarding fossil woods, only two have been discovered in localities of Peru. Pons and De Franceschi [34] described a wood specimen from the Middle Miocene Pebas Formation and suggested one of the studied woods resembled *Anacardium*. Unfortunately, the description of this specimen is neither detailed nor illustrated, and the authors did not describe the type of vessel-ray parenchyma pits, a trait that is key in the comparison of this genus. [8] described a new fossil species of *Anacardium* (*A*. *incahuasi*) from the Early Eocene of the Fossil Forest Piedra Chamana in Peru. This wood shares several features with *Anacardium*, including the absence of growth rings, vessel density, simple perforation plates, vessel-ray parenchyma pits with reduced borders, paratracheal parenchyma, and the presence of large prismatic crystals. Although we agree that *A*. *incahuasi* shares several characters with members of the Anacardiaceae family, the vessel-ray pitting does not resemble the patterns observed in the cashew genus. We note that the patterns observed in *A*. *incahuasi* are similar to those of a new fossil genus, *Llanodelacruzoxylon sandovalii* Rodríguez-Reyes, Estrada-Ruiz et Gasson [12], which was discovered in the same Santiago Formation as the *Anacardium* described here. This new fossil wood collected in Los Boquerones, Veraguas, Panama has a distinct set of features that match the *Anacardium* genus more closely. Therefore, we conclude that it can be confidently identified as a new fossil *Anacardium* species of, named *A*. *gassonii* Rodríguez-Reyes, Estrada-Ruiz et Terrazas.

### Biogeographical significance of this new fossil *Anacardium* species

Anacardiaceae is a cosmopolitan plant family currently found in temperate, seasonally dry tropical forests and tropical wet forest regions [35]. Anacardiaceae originated in South East Asia during the Upper Cretaceous [9, 35]. By the Paleocene, the family diversified in Southeast Asia and expanded its geographic range to sub-Saharan Africa; later, Anacardiaceae colonized South America. The most recent studies suggest that Anacardiaceae dispersed into North America, Oceania, and Madagascar, with some ancestors from tropical wet climatic niches expanding into tropical dry as well as temperate climatic regions [9].

Given the distribution of extant Anacardiaceae, long distance dispersal and vicariance events appear to be the most likely explanation for the modern distribution of the family [9, 36, 37]. Currently, Anacardiaceae is not found in extreme cold regions in the Northern Hemisphere; however, during the Paleocene Eocene Thermal Maximum (PETM) many tropical species spread throughout those regions [9, 38, 39].

**Fig 5:**
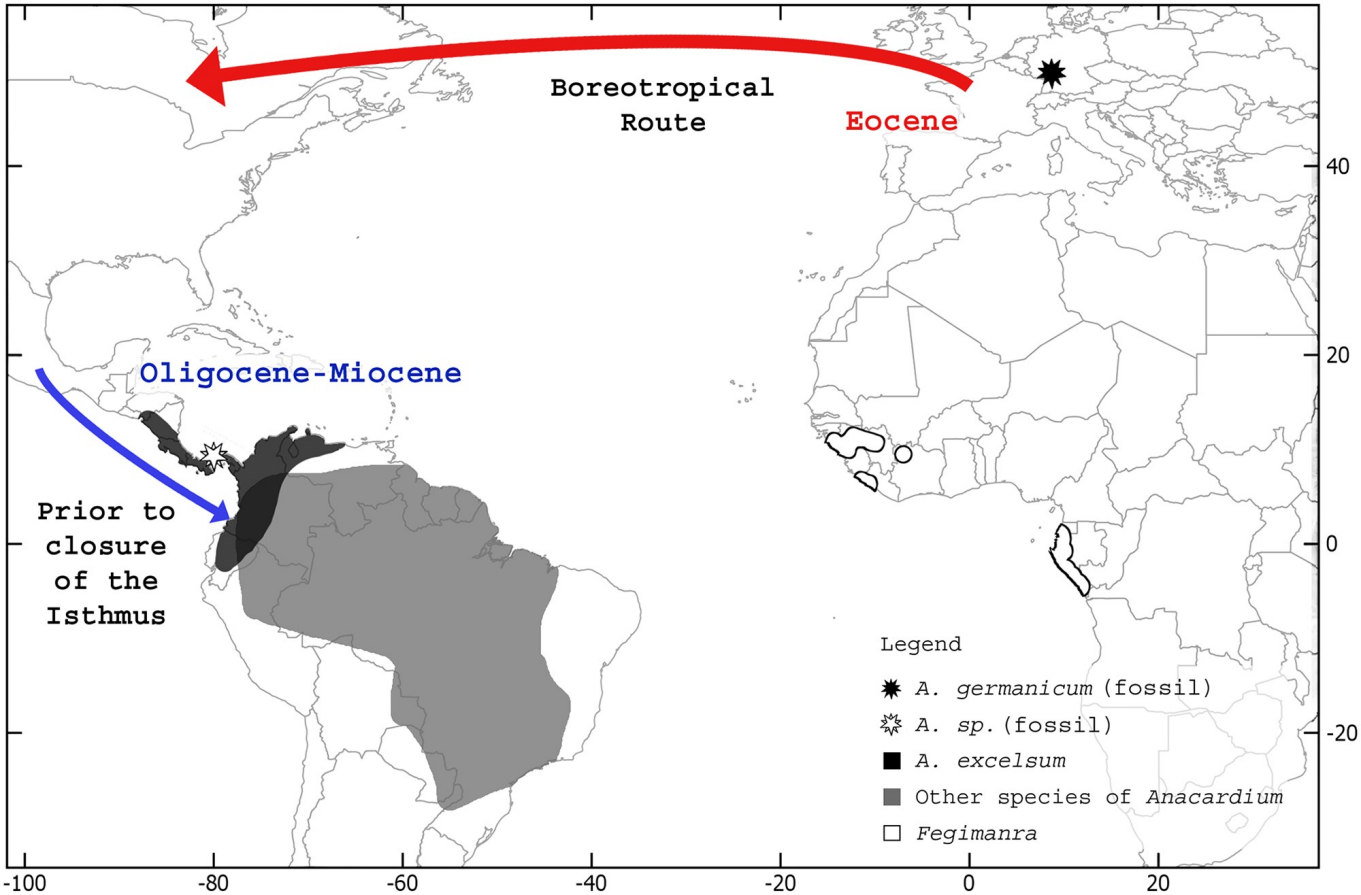
Map illustrating hypothetical migration routes for *Anacardium* showing the modern distribution of *Anacardium* and *Fegimanra*. and the occurrence of *A*. *germanicum* in Germany and *A*. *gassonii* in Panama.

Within Anacardiaceae, the genus *Anacardium* is restricted to the Neotropical region. This location is contrary to its sister group *Fegimanra* Pierre, a paleotropical genus that is restricted to the West African coast, and other phylogenetically close genera (e.g., *Bouea* Meisn., *Mangifera* L., *Swintonia* Griff. and *Gluta* L.) also native to paleotropical continents [24, 35]. Three *Anacardium* species are found in South American savannas, whereas the rest of the species occur in humid forests [25]. *Anacardium excelsum* is the only species within *Anacardium* that occurs in the northern region of the Andes and in Central America. The current distribution of this species, together with the anatomical and geographic proximity to *A*. *gassonii*, may indicate a relict occurrence of the genus in those regions prior to the closure of the Central America Seaway (Fig 5).

The earliest reliable evidence of the cashew genus is a fossil fruit named *Anacardium germanicum* from the Eocene of Germany [14]. This fossil shows that *Anacardium* was widespread in the Eocene and suggests a paleotropical origin, as well as for other taxa in the family. The occurrence of *A*. *germanicum* in Messel also supports the hypothesis of a Boreotropical route from Eurasia to North America during warm climatic intervals and the subsequent colonization of tropical areas in Central and South America [14].

Past migrations that were facilitated by changes in continental arrangement and climatic changes in temperate regions from the Northern Hemisphere (Boreotropical vegetation) are well known in other genera. For example, *Rhus* L. (Anacardiaceae) [40] and *Staphylea* L. (Staphyleaceae) [41] have disjunct distributions between Asia, Europe, and the Americas, while *Searsia* F.A. Barkley (Anacardiaceae) [42] is found in southern Africa and Asia.

The discovery of *A*. *gassonii* confirms that the genus was present in the neotropics during the Oligocene-Miocene. This new fossil species, the distribution of extant *Anacardium* species, and the last proposed phylogenetic dating [9] support previous conclusions regarding migration routes and allow us to infer that the genus crossed the Central American Seaway (CAS) prior to its final closure (Fig 5) [17].

## Conclusions

*Anacardium gassonii* shares most features with *Anacardium excelsum*, including the presence of large vessels, a lozenge-aliform parenchyma pattern, large and polygonal intervessel pitting, simple vessel-ray pitting slightly in palisade, and abundant large prismatic crystals in upright and square ray cells. The identification of this new species strongly supports the occurrence of the *Anacardium* genus in Central America during the Oligocene-Miocene and adds to previous conclusions regarding the rainforests that dominated this region prior to the closure of the Panama Isthmus. This discovery also adds an important link to the historical migration patterns of the genus, supporting the idea of an Eocene migration to the Neotropics via Boreotropical bridges, and subsequent crossing of the CAS during the Oligocene-Miocene leading to diversification in South America.
